# DKK1 and Its Receptors in Esophageal Adenocarcinoma: A Promising Molecular Target

**DOI:** 10.3390/diagnostics15010085

**Published:** 2025-01-02

**Authors:** Markos Despotidis, Orestis Lyros, Tatiana S. Driva, Panagiotis Sarantis, Emmanouil I. Kapetanakis, Adam Mylonakis, Andreas Mamilos, Stratigoula Sakellariou, Dimitrios Schizas

**Affiliations:** 1 First Department of Surgery, National and Kapodistrian University of Athens, Laikon General Hospital, 11527 Athens, Greece; adam.mylonakis@gmail.com (A.M.); schizasad@gmail.com (D.S.); 2 Fourth Department of Surgery, Attikon University Hospital, National and Kapodistrian University of Athens, 12462 Athens, Greece; lyrosorestis@gmail.com; 3 First Department of Pathology, Medical School, National and Kapodistrian University of Athens, 11527 Athens, Greece; tatianadriva@gmail.com (T.S.D.); sakellarioustrat@yahoo.gr (S.S.); 4 Department of Biological Chemistry, School of Medicine, National and Kapodistrian University of Athens, 11527 Athens, Greece; panayotissarantis@gmail.com; 5 Department of Thoracic Surgery, Attikon University Hospital, National and Kapodistrian University of Athens, 12462 Athens, Greece; 6 Institute of Pathology, University of Regensburg, 93053 Bavaria, Germany; andreas.mamilos@goc.com.cy; 7 Department of Pathology, German Oncology Center, Limassol 4108, Cyprus

**Keywords:** esophageal adenocarcinoma, DKK proteins, DKK receptors, Wnt signaling pathway, molecular target

## Abstract

Esophageal adenocarcinoma (EAC) is an aggressive gastrointestinal (GI) malignancy with increasing incidence. Despite the recent progress in targeted therapies and surgical approaches, the survival rates of esophageal adenocarcinoma patients remain poor. The Dickkopf (DKK) proteins are secretory proteins known mainly as antagonists of the Wnt/β-catenin signaling pathway, which is considered an oncogene. However, it has been shown that in several GI cancers, including esophageal cancer, DKK1 may act as an oncogene itself through Wnt-independent signaling pathways. LRP5\6 and Kremen1/2 (Krm1/2) are transmembrane receptors to which the DKK proteins are mainly known to bind. CKAP4 (cytoskeleton-associated protein 4) is a novel receptor of DKK1, and the DKK1-CKAP4 pathway seems to be crucial in the role of DKK1 as an oncogene. The aim of this review is to feature the essential role of DKK1 and its receptors in carcinogenesis with a focus on EAC in an era of urgent need for specific biomarkers along with improved targeted therapies.

## 1. Introduction

Esophageal cancer (EAC), being the eighth most common cancer and the sixth most common cause of cancer-related death worldwide [1], is characterized by poor prognosis and often delayed diagnosis in the western world; gastroesophageal junction (GEJ) adenocarcinoma incidence kept increasing during the last decades [2]. Despite recent progress in oncological management, including multimodality treatment, targeted therapies as well as modern surgical procedures, the survival rate of esophageal cancer patients remains low [3]. Recent advances in oncological therapies for solid tumors dictate a more biomarker-mediated approach rather than a histology-driven approach. In the case of esophageal adenocarcinoma, there are still no biomarkers in the clinical setting. Thus, for patients with esophageal cancer, we are still in urgent need of discovering potential biomarkers with therapeutic relevance. Therefore, we should emphasize understanding the molecular pathways that dictate the growth and the metastatic potential of the EAC tumor cells.

The Wnt signaling pathway participates in various stages of embryogenesis and the regulation of various stages of the cell cycle, while its dysregulation has been associated with carcinogenesis in various tumor types. Wnts are secreted glycoproteins with a complex signaling role. Under normal conditions, Wnt glycoproteins lead to dephosphorylation of β-catenin and its subsequent translocation to the nucleus, where it binds to the transcription factors TCF\LEF-1, resulting in the expression of target genes such as Cyclin D1, Axin2, c-MYC, and DKK1 [4].

The Dickkopf (DKK) protein family is one of the five protein families known to act as extracellular Wnt antagonists. It consists of the Dickkopf-1 (DKK1), Dickkopf-2 (DKK2), Dickkopf-3 (DKK3), and Dickkopf-4 (DKK4) proteins, and the Soggy protein (Dkkl1) [5].

**Figure 1:**
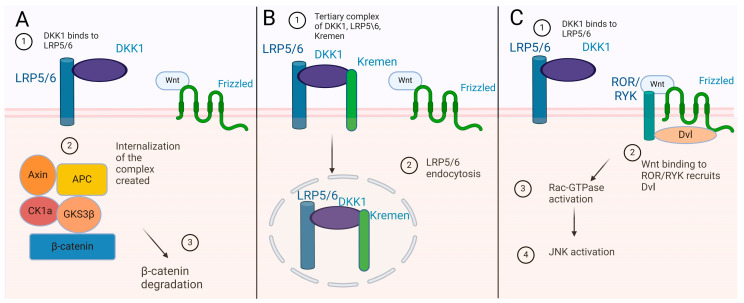
The role of DKK1: (**A**) DKK1binding to LRP5/6 leads to β-catenin degradation, (**B**) tertiary complex of DKK1 with LRP5/6 and Kremen lead to its internalization; and (**C**) DKK1 binding to LRP5/6 can lead to JNK activation independently of β-catenin. Axin, axis inhibition protein; APC, adenomatous polyposis coli; GSK-3β, glycogen synthase kinase 3β; CK1α, casein kinase 1α; ROR/RYK, receptor-like tyrosine kinase; Dvl, Disheveled; JNK, Jun N-terminal kinase.

DKK proteins are secreted proteins that bind to the transmembrane receptors KREMEN 1 (Krm1) and KREMEN 2 (Krm2) but also to the receptors LRP5 and LRP6. The binding of the DKK1 protein to the LRP5/6 receptors, which are also Wnt co-receptors, leads to the internalization of the complex that is created and, as a consequence, it causes changes in the dephosphorylation of β-catenin [4,5,6] (Figure 1A). Krm2 also seems to form a tertiary complex with DKK1 and LRP6 that leads to LRP6 endocytosis and, thus, inhibition of the b-catenin pathway [5] (Figure 1B). Additionally, the binding of DKK1 to LRP5\6 could also induce Wnt–Frizzled interactions with receptor-like tyrosine kinase, ROR/RYK, which leads to activation of Rac GTPase and consequently JNK activation through the Disheveled (Dvl) protein [7] (Figure 1C).

Despite the fact that the Wnt/β-catenin signaling has been known to drive tumor growth, the members of the DKK family that act as antagonists can also function as tumor inducers [4,8,9] (Table 1).

Taking this fact into account, the DKK1 function seems to be dictated by the receptor setting of the tumor cells. DKK1 will function according to the receptor it will find. Therefore, it is interesting to identify novel receptors that work as the binding receptor for DKK proteins. In addition, immunotherapy is a game changer in oncological treatments, and the role of DKK proteins as an immunomodulator has been supported in several reports. Therefore, it is crucial to review the role of DKK proteins in the tissue response to immunotherapy and reflect on it in the setting of EAC.

The aim of this manuscript is to summarize the current knowledge and the relation between DKK proteins and esophageal adenocarcinoma and discuss future perspectives for the development of novel biomarkers and treatments.

## 2. DKK and EAC

### 2.1. DKK Proteins Expression Levels in EAC

DKK1 has been studied both in human specimens and cell lines of esophageal adenocarcinoma [4,36,37,38]. Darlavoix et al. have proved that DKK1 is significantly highly expressed in high-grade dysplasia and EAC compared to Barret esophagus or low-grade dysplasia [36]. DKK1 upregulation was associated with the miR-33a-5p downregulation in esophageal cancer cell lines as well as in human specimens [38]. Lyros et al. have also supported that DKK1 is overexpressed in EAC human specimens and cell cultures of esophageal adenocarcinoma [4,37] and provided evidence that the DKK1 upregulation is enhanced during the sequence of esophageal metaplasia–dysplasia–EAC [37].

On the other hand, the expression of DKK2 was found to be decreased in esophageal adenocarcinoma (EAC) cell lines and even more so in 5-FU-resistant EAC cell lines [39]. Interestingly, DKK2 expression has been correlated with survival outcomes, depending on neoadjuvant treatment status. In patients who did not undergo neoadjuvant treatment, higher DKK2 expression was associated with improved survival. Conversely, in patients who received neoadjuvant treatment, higher DKK2 expression was correlated with worse survival [40]. These findings suggest a dual role of DKK2, possibly influenced by the tumor microenvironment and therapeutic interventions. Additionally, DKK3 expression was found to be elevated in 116 human specimens of EAC but was absent in the EAC cell lines studied [41].

### 2.2. DKK Proteins Function in EAC

DKK1 was reported to favor tumor growth and metastatic state in EAC. Upregulation of DKK1 in EAC cell lines enhances cancer cell proliferation as well as cell invasion and migration [4]. Reversely, DKK1 knockdown appeared to have a negative impact on cancer cells, proliferation, and migration by enhancing apoptosis [4,38]. Interestingly, this tumor-inducing function of DKK1 in cell lines of esophageal adenocarcinoma was shown to be independent of b-catenin as opposed to esophageal squamous cell lines and Barret esophagus cell lines [4,37]. Consequently, the oncogenic function of DKK1 appears to be Wnt-independent.

Nonetheless, DKK1 was downregulated in negative lymph nodes of patients with esophageal cancer and pN1 compared with negative lymph nodes of pN0 patients [42]. This fact implies that there may be pre-metastatic transcriptional changes in lymph nodes, while it highlights the crucial role of the microenvironment in the function of DKK1.

Similarly, when DKK3 was transferred to EAC cell lines, proliferation, invasion, neoangiogenesis, and chemoresistance were enhanced, while DKK3 overexpression in human specimens was correlated to nodal metastases and pathological stage [41].

### 2.3. DKK Proteins as Serum Biomarkers in EAC

In the base of DKK1 overexpression in EAC human specimens, Ramirez et al. studied the levels of serum DKK1 in patients with cancer of the esophagus. The serum DKK1 was increased in EAC patients in comparison with the control group, while EAC patients had lower levels of serum DKK1 after neo-adjuvant treatment than those without any treatment prior to surgery. Remarkably, both recurrence rates and median overall survival (OS) were correlated with the serum levels of DKK1 prior to surgery [43]. Another study also demonstrated that higher serum DKK1 levels correlate with higher TNM-staging as well as worse 5-year OS [38]. These data suggest that DKK1 could be a very useful serum biomarker in the clinical setting, and it could be used in decision-making in order to identify high-risk patients with worse tumor biology.

## 3. DKK Proteins in Correlation with Other Receptors Studied in EAC

### 3.1. EGFR—Her2

The epidermal growth factor receptor (EGFR) and Her2 are members of the ErbB family of receptors, a subfamily of four closely related receptors of tyrosine kinases: EGFR (ErbB-1), Her2/neu (ErbB-2), Her 3 (ErbB-3), and Her 4 (ErbB-4), whose activation is associated with increased cell proliferation, differentiation, and metastasis.

In EAC, EGFR has been found to be highly expressed and its expression to be associated with increased tumor invasion (pT). EGFR gene amplification has also been associated with a higher stage of the disease, less time to recurrence, and less cancer-specific survival [44]. At the same time, EGFR gene amplification was reported to be more prevalent in the distal esophagus and GEJ cancer than in gastric cancer [44]. Accordingly, EGFR overexpression led to shorter OS, too [45,46], while its expression has been associated with worse pathologic features such as pT, venous and lymphatic invasion, and lymph node metastasis in GEJ adenocarcinomas [47]. Of note, EGFR expression has been found to be altered during the progression from gastroesophageal reflux disease to Barrett esophagus, high-grade dysplasia, and EAC [48,49]. However, till now, there has been no clinical benefit with anti-EGFR treatment in EAC [50], signifying crosstalk signaling pathways that could explain the escaping of EGFR blockage downstream activation.

Her2, also a member of the EGFR family, is also overexpressed in EAC [51,52,53] and has been correlated to the progress from Barrett to EAC [52]. As shown in the ToGA clinical trial, the addition of trastuzumab, a recombinant humanized monoclonal antibody against Her2, to chemotherapy led to improved survival in HER2-positive advanced GEJ adenocarcinoma [54]. Since then, in several clinical trials, trastuzumab has shown efficacy against EAC in combination with chemotherapy, immunotherapy, or bevacizumab in neoadjuvant, adjuvant, or perioperative settings [55,56,57,58,59]. However, other specific anti-Her2 molecules, such as lapatinib, Trastuzumab emtansine (T-DM1), or dual anti-Her2 therapy (pertuzumab and trastuzumab), have not demonstrated similar efficacy against esophageal adenocarcinoma [60,61,62], dictating a more complex downstream signaling pathway activation.

DKK1 overexpression in lung adenocarcinoma has been reported to promote tumor growth and, interestingly, has been linked to resistance to anti-EGFR therapy [63], allowing speculation on a possible role of DKK1 in EGFR downstream activation. The immune status and the expression of immune checkpoints were also correlated with a higher risk of resistance [63], and consequently, DKK1 could promote treatment resistance by acting as an immunomodulator. On the other hand, in breast cancer, cancer cells can develop trastuzumab resistance by downregulating DKK1 and activating the Wnt signaling pathway [64]. To extrapolate, a possible correlation of DKK1 expression with Her2-positive expression in EAC might provide an explanation of Her2 drug resistance or reveal a subgroup of patients that are more prone to respond to target therapies. Such scenarios merit further investigation. However, such a correlation between EGFR or Her2 and DKK1 still needs to be investigated in EAC.

### 3.2. VEGF—VEGFR-2

Vascular endothelial growth factor (VEGF) has been found to be highly expressed in EAC and GEJ adenocarcinomas [65,66,67], while it was correlated to tumor invasion, too [67]. Serum VEGF was also higher in patients with EAC and is related to worse OS and worrisome pathologic features [68]. However, bevacizumab, a monoclonal antibody against VEGF, did not manage to show a significant effect in EAC [69,70]. In contrast, Ramucirumab, a monoclonal antibody that also acts as a VEGFR-2 antagonist, was found to improve overall survival as well as disease control when used either as monotherapy or combined with chemotherapy as second-line treatment in phase III trials [71,72]. Despite the fact that no such efficacy was proved when used as first-line treatment in patients with metastatic tumors [73], Ramucirumab plus FLOT as a perioperative treatment showed promising efficacy [74]. Apatinib, another molecule that inhibits both VEGFR and Her2, has also shown efficacy in advanced EAC [75]. Those therapeutic effects signify a role for VEGFR in EAC tumor biology, which has, until now, not been clarified.

Expression of DKK1 has been positively correlated with the expression of VEGF in various cancer types. Lower expression of DKK1 in colorectal cancer downregulated VEGF [10], while higher expression of DKK1 was associated with higher expression of VEGF in hepatocellular carcinoma [76] and cholangiocarcinoma [77]. These observations underline the importance of further investigation of the role of DKK proteins in conjunction with VEGFR-mediated pathways in EAC.

### 3.3. c-MET

c-MET, the hepatocyte growth factor (HGF) receptor, is upregulated in EAC, and it is associated with poorer prognosis and OS [78,79]. Crizotinib, an oral inhibitor of several kinases that inhibits c-MET, too, has shown promising preliminary results when used in patients with upregulated c-MET in phase I studies [80,81]. Nonetheless, the addition of various c-MET inhibitors to chemotherapy did not lead to an improved clinical response in phase II [50,82,83] or III clinical trials [84]. In lung cancer, particularly those with c-Met activation, the pharmaceutical blockade of c-Met resulted in reduced tumor growth via suppression of DKK1 expression [85]. Although no data exists at present, the possible correlation between c-MET and DKK1 in EAC is an attractive field of research.

## 4. DKK as Immunomodulator

Activation of the Wnt signaling pathway participates in the function of immune cells and T cell and B cell development. In fact, DKK1 has been shown to contribute to the modulation of various immune cell activities, resulting in the enhancement of tumor progression [86]. DKK1 seems to have immunomodulatory effects in the tumor microenvironment, inducing immunosuppression and a low anti-tumor response by impeding a Natural Killer (NK) cell-mediated response and enhancing myeloid-derived suppressor cell activity [87]. DKK1 seems also to affect PDL-1 expression [87,88].

Programmed cell death protein 1 (PD-1) is a receptor on the T cell surface that suppresses lymphocyte activation and immune cell function. Programmed cell death ligand 1 (PDL-1) is overexpressed in various cancers, among them also in esophageal adenocarcinomas, especially in those with microsatellite instability (MSI) [89,90]. In EAC, PDL-1 is also expressed in lymph node metastasis as well as in distant metastasis, and it can be used as a prognostic factor, too [91,92,93,94]. Immunotherapy regimens that bind to PD1 and inhibit ligation to PDL-1 have been approved as treatment options in EAC [95].

Latency-competent cancer (LCC) cells seem to play an important role in cancer progression and metastasis. LCC cells can express DKK1, a transcriptional target of Sox2, to enter a slow-cycling state and act like stem cells. Overexpression of DKK1 results in the downregulation of NK cell-activating ligands and impedes NK cell-mediated clearance of the LCC cells as well [86,96].

Furthermore, DKK1 expression is positively correlated with the accumulation of myeloid-derived suppressor cells (MDSCs) in cancer. Elevated levels of DKK1 also decrease the number of CD8^+^ T cells, CD45^+^ leukocytes, and NK cells and impede T cell activation and proliferation [86,97].

DKN-01, an anti-DKK1 antibody, has been studied in combination with pembrolizumab in advanced esophagogastric cancer in a phase I study [87], as well as in combination with tislelizumab [98,99], with promising preliminary results. DKN-01 also plays an immunomodulatory role, demanding the presence of NK cells as well as the unimpeded function of the immune system in order to enhance its anti-tumor activity [100]. At the same time, DKN-01 can lead to the downregulation of MDSCs and upregulation of CD45+ leukocytes [86].

In gastric cancer, inhibition of DKK1-enhanced NK group 2 member D (NKG2D) CAR-T cells’ function by elevating the number of NKG2D ligands [101]. Moreover, in colorectal cancer with impaired mismatch repair, inhibition of DKK1 improved the response to anti-PD-1 therapy by enhancing CD8^+^ T cell proliferation and function [102].

One of the reasons for inadequate PD-1 responsiveness is the expansion of intra-tumoral MDSCs, which is enhanced by high levels of DKK1. In addition, anti-DKK1 therapy can induce PDL-1 expression on intra-tumoral MDSCs as well as reduce their number [103]. This explains why the combination of anti-DKK1 and anti-PD/PD-L1 therapy seems to have encouraging preliminary results, especially in patients with high expression of DKK1 [87]. Consequently, the combination of anti-DKK1 and anti-PD/PDL-1 therapy could be a possible hallmark in cancer treatment [86,102].

DKK3 seems also to have a crucial role as an immunomodulator. In fact, DKK3 participates in the maturation of B cells, the tolerance of CD8^+^ T cells, and the differentiation of monocytes to dendritic cells [104]. In various cancers, DKK3 may act as an oncogene through immunomodulation of the tumor microenvironment [104], and it could also explain its oncogenic function in EAC (41).

## 5. Clinical Trials of DKK1 in EAC

DKK1 has also been studied as a potential target of anti-cancer therapy. DKN-01, an IgG4 monoclonal antibody against DKK1, is being studied in trials as monotherapy or combination therapy in advanced GEJ adenocarcinoma [98] (Table 2).

DKN-01 as monotherapy or in combination with paclitaxel or pembrolizumab in esophagogastric cancer (EGC) has been studied in a phase Ib study (NCT02013154). In preliminary results, the combination of DKN-01 plus paclitaxel demonstrated sufficient anti-tumor activity with a favorable safety profile [105]. At the same time, DKN-01 in combination with pembrolizumab showed a favorable safety profile, while the anti-tumor efficacy was superior mainly in anti-PD-1/PD-L1-naïve patients with high expression of DKK1 [87].

Another phase 2a study of DKN-01 in combination with tislelizumab, an anti-PD-1 antibody, plus CAPOX chemotherapy in Her2-negative advanced gastric or gastroesophageal junction adenocarcinomas (DisTinGuish) showed prolongation of PFS and OS with a favorable safety profile. Among 25 patients receiving first-line treatment, 21 had dictated levels of DKK1, while 22 had dictated levels of PD-L1, with the majority having a low expression of PD-L1, though [98]. DKN-01, in combination with tislelizumab, displayed promising efficacy as a second-line treatment in patients overexpressing DKK1. The most promising and durable results were noticed in DKK1 high/PD-L1 high (vCPS ≥ 10) patients [99]. After these results, in ongoing part C of DisTinGuish trial, 160 Her2negative patients with unresectable, locally advanced, or metastatic disease who have received no prior therapy will be randomized to a 1:1 ratio to receive either DKN-01 in combination with tislelizumab and chemotherapy regimen (CAPOX or mFOLFOX6) (*n* = 80) or tislelizumab in combination with a chemotherapy regimen (CAPOX or mFOLFOX6) alone (*n* = 80) [NCT04363801].

Furthermore, DKN-01 is currently studied in combination with atezolizumab in patients with advanced gastroesophageal cancer who have progressed following chemotherapy in a phase II clinical trial [NCT04166721].

## 6. Potential Receptors for DKK-Mediated Function in EAC: A Novel Interpretation

DKK proteins are known as Wnt antagonists, but it seems that they can also act outside the ordinary Wnt pathway [40]. CKAP4 (cytoskeleton-associated protein 4) has also been proven to be a DKK1 receptor [6,106] and could play a crucial role in this pathway [107,108]. CKAP4 (also named P63, CLIMP-63, and ERGIC-61) is mainly a protein of the endoplasmic reticulum (ER) contributing to the stabilization of ER. A small amount of CKAP4 can also be transferred to the cell surface through the trans-Golgi network, where it acts as a type II transmembrane protein [107,108,109]. In contrast with LRP and Kremen, CKAP4 binds to DKK1 through CRD1, which is a more conserved domain than CRD2 in the DKK family, and as a result, other members of the DKK family could also be ligands of CKAP4 [5,6,106]. In fact, although DKK3 is mainly known as a tumor suppressor, it also binds to CKAP4 through CRD1, acting as an oncogene [110].

**Figure 2:**
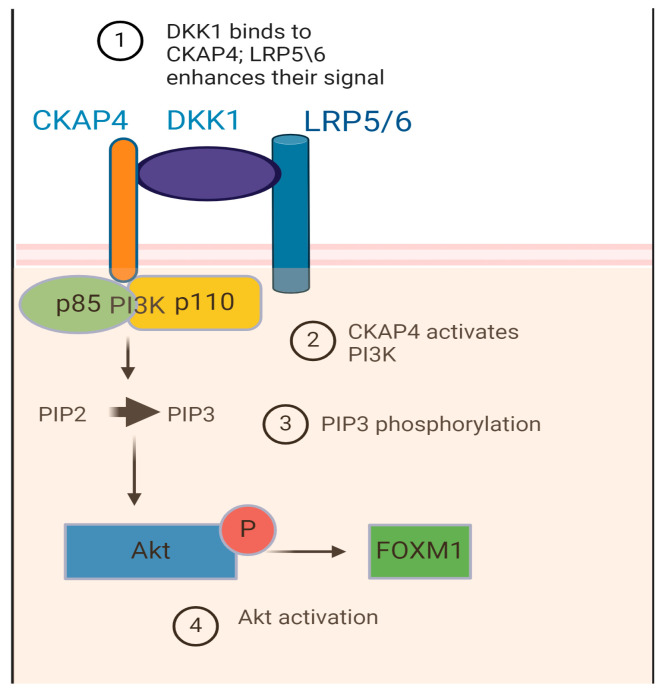
DKK1-CKAP4 axis. The presence of both DKK1 and CKAP4 leads to activation of Akt-mediated oncogenic molecular pathways. CKAP4, cytoskeleton-associated protein 4; PI3K, phosphoinositide 3-kinase (consisted of p85 and p110 subunits); PIP2, phosphatidylinositol (4,5)-bisphosphate; PIP3, phosphatidylinositol (3,4,5)-trisphosphate; Akt protein kinase B; FOXM1, forkhead box M1.

After binding to DKK1, CKAP4 activates PI3K (phosphoinositide-3-kinase) through a proline-rich domain, which in turn phosphorylates PIP3 (phosphatidylinositol(3,4,5)-triphosphate) resulting in the activation of intracellular signaling pathways mediated by Akt (protein kinase B) [6,107,109] (Figure 2).

In these pathways, the process of palmitoylation seems to play an important role, as it contributes to the transfer of LRP6 from ER to the cell surface and CKAP4 to the lipid rafts of the cell membrane. Additionally, a ternary complex among DKK1-LRP5\6-CKAP4 seems to enhance the proliferative DKK1-CKAP4 signal [108,109]. DKK1 controls and induces depalmitoylation of LRP5\6 and CKAP4, leading to endocytosis and inactivation of its own receptors [109].

Apart from the various biological processes in which CKAP4 is involved, it also acts as a pro-tumor molecule. It has been shown that binding of DKK1 to CKAP4 leads to cell proliferation, while anti-CKAP4 antibodies seem to have anti-tumor activity [106]. The necessity of the expression of both DKK1 and CKAP4 in tumorigenesis is highlighted by many studies [6,107,111]. DKK1 and CKAP4 were highly expressed in lung adenocarcinoma, pancreatic duct adenocarcinoma as well as esophageal squamous cell carcinoma and, apart from this, the synchronous expression of DKK1 and CKAP4 was correlated to a worse prognosis [6,31,111,112], while other CKAP4 ligands were not involved in cell proliferation of those cancers [6]. Additionally, the use of an anti-CKAP4 antibody or a DKK1 that was unable to bind to CKAP4 impeded EAC cell line growth [110,111,112]. CKAP4 can enhance cell migration independently of DKK1 as well. CKAP4 binding to β1 integrin induces the recycling of α5β1 integrin, and consequently, it reduces the stability of cell adhesion [113].

Given the above, a possible explanation of the diverse roles of DKK proteins in carcinogenesis lies in the biological context in which they are expressed. In fact, we should not interpret the function of DKK proteins as units but in combination with the expression of their receptors in each microenvironment. We should bear in mind that they can act either in a Wnt-dependent manner or in a Wnt-independent manner.

The DKK-CKAP4 axis seems to be involved in the tumorigenesis of EAC independent of Wnt, and it could be a promising molecular pathway that could elucidate the role of DKK1 in EAC even more. DKK1 is increased in tumor specimens of patients with EAC [4,36,37,38]. Additionally, the synchronous expression of both DKK1 and CKAP4 seems to result in a worse prognosis in different types of cancer [6,107,111,112]. Consequently, it seems that the presence of both of these molecules may be the key to their oncogenic function.

DKK3 can also be found increased in EAC [41]. DKK3 was shown to enhance tumorigenesis through the TGFβ pathway, but both Kremen1/2 and CKAP4 can be receptors of DKK3 [41,114]. Although little is known about the role of the other members of the DKK family in EAC, all of them appear to bind to CKAP4 and Kremen1/2 [5,41]. A potential role for them in the carcinogenesis of EAC can be hypothesized.

Although it has not been studied in the serum of patients with EAC, CKAP4 is also secreted into the serum and has been found elevated in various types of cancer. It could, therefore, be used as another serological cancer biomarker [115]. Serum DKK1 is highly elevated in EAC [38,43]. These molecules could prove very useful in the early diagnosis of EAC and serve as valuable pre-therapeutic serologic markers.

Interestingly, targeting the DKK1-CKAP4 pathway could be a significant way to develop novel therapeutic agents. Knockdown of DKK1 or CKAP4 inhibited cell proliferation [4,112], and the anti-CKAP4 antibody seems to suppress tumor formation [112]. Last but not least, DKK1 can elevate PD-L1 expression in a CKAP4-dependent way through the activation of PI3K-Akt signaling [88]. Thus, either DKK1 or CKAP4 could serve as molecular targets for anti-cancer therapy. Several molecules have been studied as anti-DKK1 drugs, and DKN-01 has shown some promising results [87,98]. An anti-CKAP4 antibody, an mi-RNA, or a vaccine could be an alternative [6]. A humanized anti-CKAP4 antibody showed efficacy against pancreatic cancer in murine cancer models by inhibiting the DKK1-CKAP4 axis [116]. CKAP4 could be the ligand to which more than one DKK protein binds. At the same time, CKAP4 may enhance cell migration independently of DKK1 [113]. Finally, the combination of such a targeted therapy with immunotherapy could be a cutting-edge point in the treatment of EAC.

Given the fact that the majority of EAC patients with locally advanced EAC undergo pre-surgical treatments, neoadjuvant regiments could modify the tumor molecular microenvironment. This modulation in the tumor microenvironment could be a reason for poor or inadequate responsiveness in treatment. Expression of DKK2 was shown to be decreased in 5-FU-resistant EAC cell lines [39] as well as correlated with worse survival in patients having received neoadjuvant treatment [40], and DKK3 improved chemoresistance in EAC [41]. Consequently, DKK proteins and their receptors could serve as predictors of response to chemotherapy, although further research is needed in this field.

Further studies in animal models and human specimens are required to enhance our understanding of the exact role of DKK proteins and their possible receptors in EAC. Such an understanding will help the development of possible anti-cancer drugs. According to the data presented in this review, this field is really promising.

## 7. Conclusions

DKK proteins play a crucial role in EAC, and they are involved in tumorigenesis and disease progression. DKK proteins and their potential receptors could represent valuable prognostic biomarkers and therapeutic targets in EAC. Notably, the potential role of DKK as an immunomodulator could feature novel therapeutic hallmarks in EAC.

## Figures and Tables

**Table 1:** Role of DKK1 in cancer.

Type of Tumor	DKK1 Role	Downstream Mechanism	Receptor That Mediates the DKK1 Function	Studies
Colorectal cancer	1. Tumor suppressor 2. Oncogene	1. Inhibition of expression of VEGF [10], inhibition of Wnt/β-catenin pathway [11] 2. Independent of Wnt [12]	1. LRP5/6 [11], not defined [10] 2. Not defined	1. Liu et al. [10], Qi et al. [11] 2. Aguilera et al. [12], Gurluler et al. [13]
HCC	Oncogene	Wnt/β-catenin pathway [14]	Not defined	Yu et al. [14], Yang et al. [15], Tung et al. [16], Tao et al. [17]
Gastric cancer	Oncogene	Not defined	Not defined	Lee et al. [18], Liu et al. [19]
Ovarian cancer	Oncogene	Overexpression/activation of P-JNK1 [20]	Possibly Fzd [20]	Klotz et al. [21], Shizhuo et al. [22], Wang et al. [20]
Breast cancer	Oncogene	Wnt/beta-catenin pathway [23]	Not defined	Zhou et al. [24], Xu et al. [23]
Lung cancer	Oncogene	Not defined	Not defined	Dong et al. [25], Yamabuki et al. [26], Sheng et al. [27]
Esophageal carcinoma	Oncogene	Phosphorylation of Akt	Not defined	Lyros et al. [4], Yamabuki et al. [26], Begenik et al. [28], Peng et al. [29], Makino et al. [30] Kimura et al. [31]
Urothelial cancer	Oncogene	Not defined	Not defined	Shen et al. [32], Sun et al. [33]
Pancreatic cancer	Oncogene	AKT/MEK-ERK pathway, overexpression of FOXM1	CKAP4	Kimura et al. [31], Takahashi et al. [34], Liu et al. [35]

**Table 2:** Studies of DKN-01 in EAC.

Study	Phase	n	Treatment Line	Treatment Arm	2nd Treatment Arm	OS/PFS
**NCT02013154 [87,105]**	1b	22	≥2nd line	DKN-01 + paclitaxel [105]	-	28.9/17.7 (weeks)
		63	≥2nd line	DKN-01 + pembrolizumab [87]	-	20.4/6 (weeks)
**DISTINGUISH-PART A [98]**	2	25	1st line	DKN-01 + tislelizumab + CAPOX	-	19.5/11.3 (months)
**DISTINGUISH-PART B [99]**	2	52	2nd line	DKN-01 + tislelizumab	-	1.4/-(months)
**DISTINGUISH-PART C [NCT04363801]**	2	160 (1:1)	1st line	DKN-01 + tislelizumab + CAPOX or FOLFOX6	Tislelizumab + CAPOX or FOLFOX6	Ongoing
**NCT04166721**	2	Recruiting	≥2nd line	DKN-01 + atezolizumab	-	Ongoing

## Data Availability

Not applicable.
